# Acceptability and Effectiveness of COVID-19 Contact Tracing Applications: A Case Study in Saudi Arabia of the Tawakkalna Application

**DOI:** 10.7759/cureus.35041

**Published:** 2023-02-15

**Authors:** Safia Dawood, Khulud AlKadi

**Affiliations:** 1 Health Informatics, King Saud Bin Abdulaziz University for Health Sciences, Riyadh, SAU

**Keywords:** privilege, permission, tawakkalna, security, privacy, mhealth, acceptability, contact tracing, covid-19

## Abstract

Background

Contact tracing applications were introduced during the COVID-19 pandemic to mitigate the spread of the infection in several countries. In Saudi Arabia, the Tawakkalna application was developed. The Tawakkalna application is a mobile health solution aimed to track infection cases, save lives, and reduce the burden on health facilities. This study aims to explore the public’s attitude to and acceptance levels of the Tawakkalna application and to evaluate its effectiveness regarding privacy and security. The main objective of this study is to investigate the user acceptability of contact tracing applications and explore the safety and privacy effectiveness of the COVID-19 contact tracing application, the Tawakkalna application. In addition, the study analyzes factors associated with acceptance levels and compares the results obtained to similar studies in other countries using similar applications.

Methodology

This study used a valid and reliable online survey that was used in similar studies conducted in other countries to assess the acceptability of the application. The survey was conducted from September to November 2021, and the final dataset included 205 participants. To investigate the privacy and security performance of the Tawakkalna application, we followed the investigation method used by similar research that investigated 28 contact tracing applications across Europe.

Results

Out of the 205 participants, 84.87% were in favor of the opt-in voluntary installation of the Tawakkalna application, and 49.75% of the participants were in favor of the opt-out automatic installation. Individuals’ trust in the government had a huge impact on acceptance, with 60.98% of the participants supporting the application because they believed that the Tawakkalna application would help them stay healthy during the COVID-19 pandemic. Overall, 49% of the participants supporting the application also agreed to the de-identification of their collected data and providing it for research. The Tawakkalna application ranked at the top compared to other contact tracing applications regarding privacy and security.

Conclusions

The Tawakkalna application developed by the Saudi Data and Artificial Intelligence Authority was a response to the COVID-19 pandemic, which is considered the biggest public health crisis in recent times. The Saudi Arabian government gained the population’s acceptance through effective endorsement and the spread of educational content through media channels. By complying with privacy policies, the Tawakkalna application is an effective tool to combat public health infectious diseases.

## Introduction

The use of information and communication technology (ICT) has grown drastically following the innovation in the field during the last quarter of the 20th century [1]. This steady growth in ICT affects all aspects of human lives, especially healthcare, thus affecting the quality of life [2].

Countries tried to adopt different ways to battle the COVID-19 pandemic, with, most notably, lockdowns, quarantines, and self-isolation. Although these were proven to be effective to some degree, they had a devastating effect on the national and global economy and the mental health of the population [3]. Many countries have, therefore, resorted to developing various types of contact tracing applications, with several countries launching them as soon as they were ready. These applications provide a form of managing the pandemic by identifying any close contact with possible positive cases of individuals carrying the virus.

Amnesty International, an independent non-profitable organization, published a groundbreaking privacy and security report in June 2020. Amnesty’s Security Lab investigative study focused on contact tracing applications in countries in Europe, the Middle East, and North Africa. The study included 11 contact tracing applications used in Kuwait, Lebanon, Norway, Qatar, Tunisia, United Arab Emirates, Algeria, Bahrain, France, Iceland, and Israel. The study ranked some of these applications as *bad* and others as *dangerous* in regard to privacy rights violations [4].

Massachusetts Institute of Technology published a study on December 16, 2020, which reviewed contact tracing applications in aspects of voluntary download and usage, data usage limitations, and if the data would be used for purposes other than public health [5]. Other evaluation factors included retention of data and if there was a time period after which data will be destroyed, minimization of data collection so the application would only collect data it stated it needs, whether the process of data collection was transparent enough and its steps were publicly available and understandable, and, finally, the technology used for tracking such as Bluetooth or GPS [5].

Literature review

Although contact tracing applications were available before COVID-19, they became more popular and necessary during the pandemic. On the battlefield against COVID-19, some were fruitful and others faced backlash. Some examples are described below.

COVIDSafe is an application launched by the Australian Federal Government on April 26, 2020. First, the public was concerned about the application knowing their whereabouts, but the Australian Government assured that the application does not use GPS capabilities and released the app’s source code on GitHub to allow the public to inspect it for themselves [6].

Williams et al. explored the public attitude toward the NHS COVID-19 contact tracing application. The qualitative study included six focus groups, each divided into two sections based on whether they accepted or did not accept the application. The groups met with the research team and discussed possible reasons for accepting or not accepting the use of the application [7]. The study concluded that participants across all groups needed more knowledge or clarification regarding the application. Privacy concerns, although evident in all groups, were very apparent in groups refusing to use the application.

Another study by Panchal and Singh, which targeted the application’s functionality and features, found that 62.1% of the 1,036 participants indicated that the application met their expectations. However, 43% of the participants found the application to be complicated for its intended usage [8].

In Belgium, the application Coronalert was developed as a result of the agreement between the federal government and the federal states. The application was made available on September 30, 2020. The main objective of the application was to identify individuals who had contact with a confirmed infection case for more than 15 minutes within 15 days. Before its official launch in September, the application went through two experimental phases, namely, a fictional and real-life setting, the latter involving almost 10,000 people. In a joint study between the University of Antwerp and Ghent University, Walrave et al. conducted an online survey that included 1,850 participants [9]. The study found that 35.1% of participants installed the Coronalert application, 7.8% did not, 3.5% had installed and then deleted it, and only 14.1% intended to continue using it. According to the study, reasons for not installing the application were ranked as follows: 31.1% did not see the advantage in using it, 29.3% had privacy concerns, and 21.0% thought the application will cause them stress [9].

In Germany, on June 16, 2020, the German government launched its COVID-19 contact tracing application called Corona-Warn-App. Similar to the Belgium application, Coronalert, the German application was intended to disturb the infection chains using Bluetooth to notify users if they came less than 1.5 m from each other for a period of 10 minutes or more. In addition, it allowed users to know if they were in contact with an infected individual in the past 14 days, providing the estimated time and distance of exposure [10].

## Materials and methods

Saudi Arabia is one of the first countries to invest in information systems and communication technologies to fight against the COVID-19 pandemic by launching the government-based Tawakkalna application. Tawakkalna is the official application approved by the Ministry of Health in the Kingdom of Saudi Arabia to limit the spread of COVID-19 and was developed by the Saudi National Information Center [11].

The application provides real-time and direct information about the number of cases of COVID-19 infections in Saudi Arabia. Tawakkalna also helps in the early detection of suspected cases if COVID-19 symptoms started to appear and allows citizens and residents to request exit permits during times of curfew imposed in some cities and neighborhoods due to the outbreak of COVID-19.

The Tawakkalna application allows the follow-up of cases of exit requests during curfew time and warns users if they approach affected or isolated areas due to an outbreak. Moreover, it is possible through the application to report suspected cases of COVID-19 to help individuals obtain the necessary medical assistance for themselves or others [12].

The Saudi Data and Artificial Intelligence Authority (SDAIA) launched the Tawakkalna application to support government efforts in confronting COVID-19 aligned with the interest of the Saudi leadership in preserving the health of its citizens and residents. The Saudi National Information Center helped develop the application by providing vast and accurate information [13].

**Figure 1:**
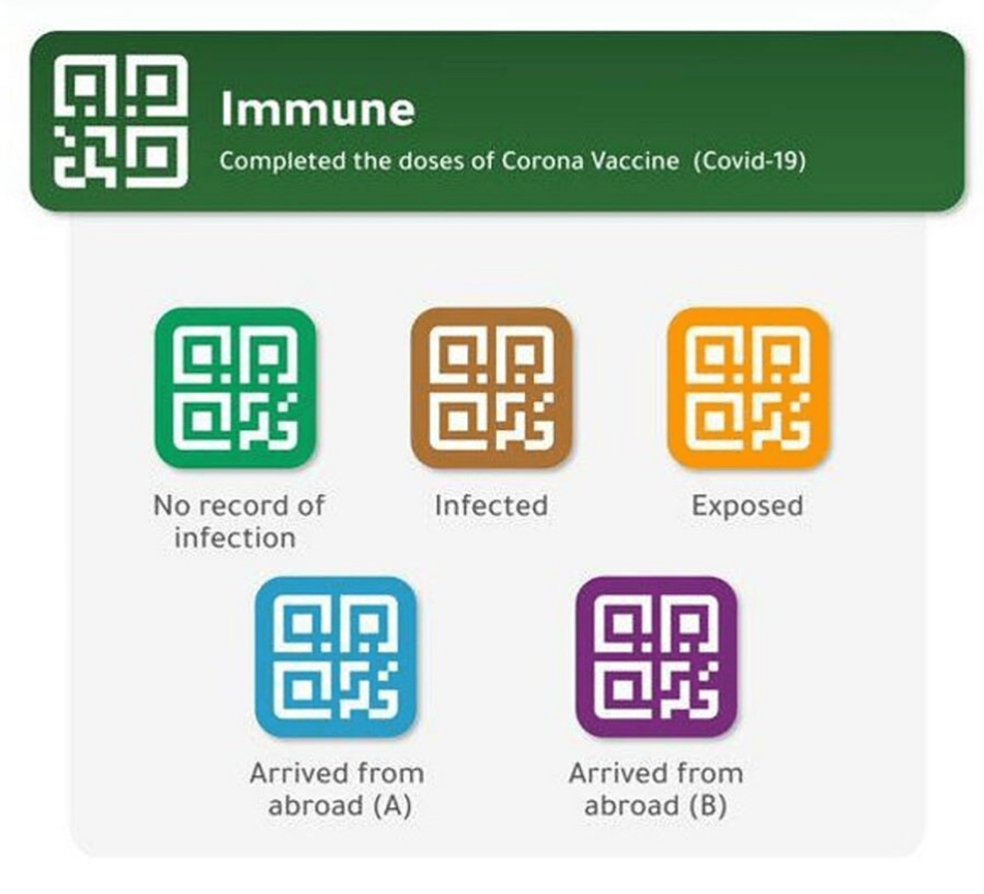
Tawakkalna application color codes. Green: no record of infection; orange color: exposed to an infected individual; brown: infected with COVID-19; blue: arrived from countries listed by the Ministry of Health as countries with high spread of infected cases; violet: arrived from countries listed by the Ministry of Health as countries with less spread of infected cases.

The Tawakkalna application was initially intended to contribute to managing the process of granting permits electronically during the curfew period. This helped limit the spread of COVID-19 in Saudi Arabia. Then, during the cautious return phase, and the lifting of the prevention measures, the application launched several important new services that contributed to achieving a safe return. Clarifying the health status of the application user through colored codes with the highest degree of safety and privacy was among the most prominent services provided, as shown in Figure 1. The application also allowed individuals to contribute to this by reporting individuals and groups violating the precautionary measures in place [14].

Moreover, through the application, it is possible to warn citizens of COVID-19 outbreaks in isolated or pandemic areas. It is also possible to obtain necessary medical assistance for any suspected case when reporting these suspected cases.

The application has undergone many development stages as it was initially launched in April 2020 to issue blocking permits. In May 2020, the application provided the health cases service represented in the health status card, and then the application allowed users to book a COVID-19 examination. Later, in January 2021, the application made it possible to obtain a health passport, and two months later, the provisions for booking COVID-19 vaccine appointments and the digital wallet were added. Finally, the capability of issuing Umrah (visiting the city of Mecca for prayers) permits and visiting the Prophet’s Mosque was added in April 2021 [15].

The Tawakkalna application provides many services during the period of return with caution, for example, the COVID-19 vaccine, health passport, mobile number identification service, as well as digital wallet. Below we introduce some of the most important services.

Umrah permits: It is a service that enables the user to view all permits adopted, for example, permits for prayer in the Grand Mosque in Mecca.

Umrah permit display service: It is a feature that allows linking with the Umrah application to display pilgrim permits in the Tawakkalna application.

COVID-19 examination service: It allows Tawakkalna users to book an appointment for a COVID-19 examination, as well as review the results of the examination through the application with ease [16].

Although contact tracing apps are helping in the current situation of the pandemic, an Oxford University study found that 60% of the UK population needs to accept, trust, and use these applications for them to be effective and allow for the full potential of their advantages [17].

Contact tracing applications have great potential to assist in managing disease outbreaks. However, the user perception and mistrust of such applications often limit their effectiveness. A study by Chen et al. indicated that people refused to download or register for such applications because they felt their privacy was being violated [18].

It is clear that the main obstacle facing contact tracing applications is the level of trust. Users have concerns such as “what will they do with my personal data?” and “Will they use my health issues against me?”. These fears need to be transparently addressed for these applications to succeed. Organizations responsible for these applications need to raise the comfort level and belief that shared personal data with the government is private and kept in secure and trustful data stores. According to YouGov reports, there is a big variation among citizens of different countries regarding governmental trust, for example, in France, about 35% of the population had trust in government applications compared to 80% in Australia in May 2020. In the US and UK, governmental trust ratings have decreased over time due to the lack of assurance and how governments use citizen data to handle crises [18].

It is natural for humans to face every new change in their lives with rejection and hesitance that can, over time, gradually turn into cautious acceptance which will end with full acceptance and endorsement [19].

The acceptance of technology, especially applications that collect sensitive data about individuals’ health is governed by factors of transparency, true beneficial and clear intentions, and by a wide spread of educational materials through the most used and trusted channels for the targeted population [20].

The Saudi government took several measures to mitigate these concerns and conducted awareness campaigns to highlight the benefits of the Tawakkalna application on the population’s health through various media channels [21]. Although this effort increased the installation rates, these rates were still very unsatisfactory to ensure strong management of the pandemic. In response, the government added another strategy to encourage people to install Tawakkalna by making it necessary to access certain services (i.e., supermarkets, malls, and government agencies). This step also contributed to a spike in new installations [22].

Methods

The purpose of this study is to explore the Tawakkalna application from the consumer’s perspective in a way that examines any limitations and allows for more desirable improvements.

A digital survey was used, which was already validated and used in previous studies [23], and was published through social media and messaging applications. The survey elements followed a five-item Likert scale and were provided in both Arabic and English, with the translation done by a professional translator. Because the application is used in Saudi Arabia, the population sample included citizens and residents who had used the application and were over 18 years old. The recruitment process utilized social media and messaging applications to spread the link to the survey.

To assess the privacy and security of the application, the study followed a method that explored four basic metrics including (i) data access permissions requests; (ii) runtime dynamic behavior in accessing resources; (iii) compliance with the guidelines of the Health Insurance Portability and Accountability Act (HIPAA) and the General Data Protection Regulation (GDPR) of Europe; and (iv) a static app-code analysis that requires access to the source code [24].

Study area/setting

Because this research examined a contact tracing application, the study focused on Tawakkalna users residing in the most densely populated regions within Saudi Arabia, including both the central and western regions.

Inclusion criteria

This study included adult users (over 18 years of age) of the Tawakkalna application residing in Saudi Arabia during the pandemic, starting from September 2021. The study examined two aspects, namely, acceptance/attitude, and efficiency regarding privacy and security through the development timeline of the application, as well as its different levels of growth in provided services since the initial launch.

Sample size

The areas of Saudi Arabia that the survey covered included Riyadh and Jeddah, which accounted for almost 43% of the targeted population, according to the World Population Review of 2021. With a confidence level of 95% and a margin error of 5%, the suggested sample size was 377 [25].

Sampling technique

Convenient sampling was used to extract a small sample from the large population of Saudi Arabia of those who met the inclusion/exclusion criteria. Furthermore, the snowball-referral sampling technique was used by including a line at the bottom of the survey asking participants to kindly pass the survey to other eligible participants; in addition, a share feature was also added for ease of distribution.

Study design

Acceptance Study Design

For acceptance and attitude, the study used a quantitative instrument published by the Journal of Medical Internet Research in August 2020 [23] and conducted the survey online in September 2021 among volunteering participants over 18 years of age. The study focused on the most densely populated regions of the Kingdom, namely, the central and western regions.

The first section of the survey was designated to elicit consent from participants and contained a brief description of the application’s purpose, functionality, and services, excluding any technical details. The second section contained three questions to measure participants’ degree of comprehension of the Tawakkalna application. The third section contained questions measuring the attitude toward voluntary installation (opt-in). This section explored the degree of willingness to install the application voluntarily, individuals’ compliance with the application’s recommendations, and reasons for or against installing the application. The fourth section contained questions measuring the attitude toward automatic installation (opt-out). The final section included demographic information about the participants, including age, gender, region, health status, and nationality. Results in Saudi Arabia were compared to those obtained in five countries (the United Kingdom, France, Germany, Italy, and the United States), where the same survey was conducted to extract lessons and conclusions [23].

Effectiveness Study Design

For effectiveness, the research explored the guidelines of HIPAA and GDPR organizations using a systematic evaluation method used by Hatamian et al., which included the evaluation of contact tracing applications used in 28 countries. The method focused on key features of contact tracing applications, including architecture, data utilization, privacy, security, and attack vulnerability [24].

Qualitative Impact Analysis

The impact analysis method published by Hatamian et al. was used. The analysis method was based on software engineering testing techniques that covered the application in different states, such as static, dynamic, and behavioral. It contained a checklist that explored application behavior that affects privacy, security, and risk standards, as well as rules and behaviors that either follow or contradict security and privacy best practices [24].

**Figure 2:**
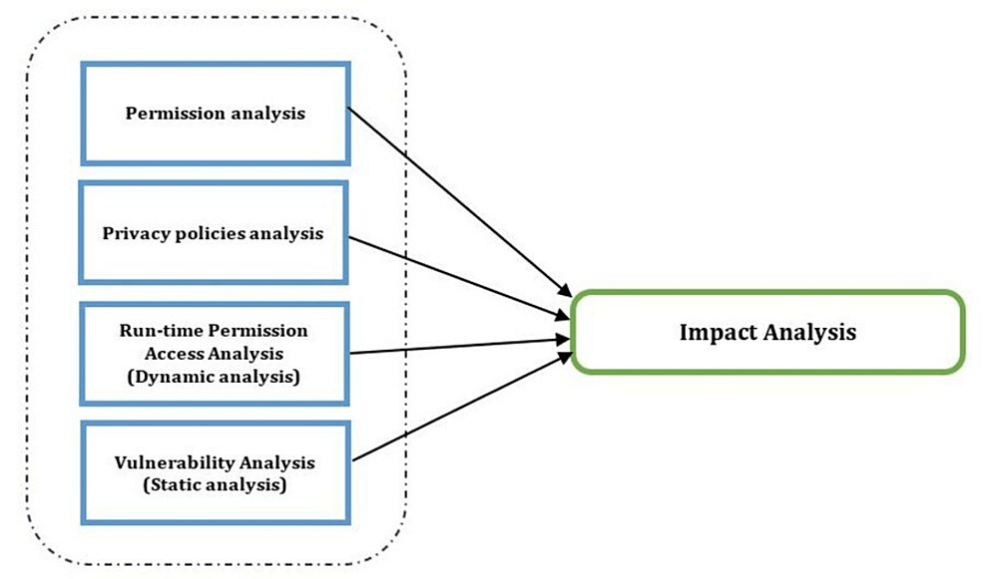
Privacy and security effectiveness analysis framework.

The privacy and security assessment technique of the contact tracing application Tawakkalna conducts an impact analysis, as illustrated in Figure 2.

Permission Analysis

Applications usually request access to users’ mobile resources through application permissions. Every time a permission is requested, the mobile user needs to grant and consent to the permission. The goal of this analysis is to check the pattern of permission requests by the application as well as the timing and type of resources required.

By observing these permissions, we can analyze application resource usage and developers’ intentions through suspicious permission requests and compare these patterns to application permission requests by other applications developed by European and non-European bodies. Android identifies three categories of permissions that this study explored for the android version of the Tawakkalna application. These permissions are Normal, Dangerous, and Signature, where Normal indicates low-risk permission requested during the application’s installation; Dangerous permissions request access to sensitive resources with high risk; and Signature permissions are requested under the application certificate.

Privacy Policy Analysis

A privacy policy is a legal statement that frames how the data collected by the application will be used, disclosed, shared, and managed by the application’s governing body. The policies require a transparent view of these practices and strict compliance with privacy and data protection legal principles. Policies must be clear and descriptive for users to understand how their sensitive data will be collected, shared, and handled by the application providers [26].

The objective of this analysis is to explore the extent to which Tawakklana complies with fundamental privacy principles and compare the findings to other applications developed by European and non-European bodies.

Run-Time Permission Access Analysis (Dynamic Analysis)

This analysis focuses on the question of how the application exercises its granted privileges to access permissions at run-time. This analysis was conducted through run-time monitoring tools, observing processes, log files, collected data, and permissions’ request patterns during run-time; for example, access to sensitive data such as GPS.

Test Bed for Monitoring Permission Usage

**Figure 3:**
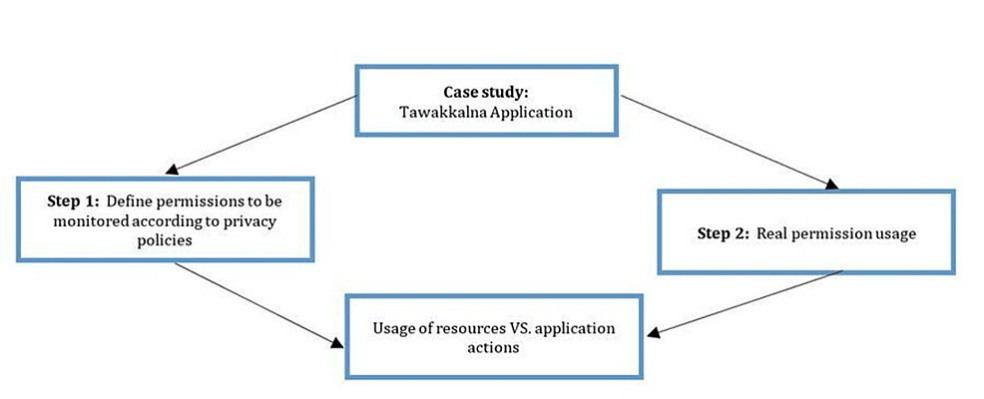
Run-time permission access analysis.

In this test, the application should have open permission and be granted access to resources whenever required. Then, an application monitoring tool is run in the background without intervening with the observed application run-time processes. The objective is to point out sensitive data collection and processing where it is not required and the time and frequency of access [27]. Figure 3 summarizes the run-time permission access analysis test.

Step 1: Defining permissions that need to be monitored that can create risks and breaches. These permissions include the following:

ACCESS_FINE_LOCATION

ACCESS_COURSE_LOCATION

ACCESS_BACKGROUND_LOCATION

CALL_PHONE

CAMERA

ACTIVITY_RECOGNITION

READ_EXTERAL_STORAGE

READ_PHONE_STATE

RECORD_AUDIO

READ_CONTACTS

PEMISSION_ACCESS_FREQUENCY

Step 2: Monitor the permission access patterns of the application. The application should be installed on several devices that have a pre-configured data capture tool. If the application fails to comply with the identified application privacy policy principles should be analyzed. Logs should be collected and analyzed and interpreted to determine application permission access patterns.

Vulnerability Analysis (Static Analysis)

In this analysis, we validated the application code files and searched for vulnerabilities using Mobile Security Framework (MobSF). MobSF is an open-source security analysis tool with the ability to detect security threats, malware, and statics/dynamic code breaches [28]. MobSF produces reports that include metrics.

Statistical analysis

The study had two principal outcome variables. One represented respondents’ willingness to install the Tawakkalna application on their mobile phones. The variable represents the installation regime opt-in and is a five-point Likert scale that was coded into binary values. The value 1 represents intentions of definitely install or probably install and 0 represents otherwise. The other variable represents the respondents’ willingness to keep the application installed on their mobile phones. The variable represents the installation regime opt-out and is a five-point Likert scale that was coded into binary values. The value 1 represents intentions of definitely keep or probably keep and 0 represents otherwise. Whereas the opt-in regime represents voluntary installation by consumers, opt-out represents automatic installation where the government forces installation through mobile phone providers.

Multiple logistic regression, one of the multivariate analysis techniques, was used to identify relationships between each of the two principal binary outcome variables with a set of independent variables, such as age, gender, nationality, education level, place of residence, health insurance, health problems (diabetes, high blood pressure, heart or breathing problems), sick pay, and trust in the government. The chi-square test was used to illustrate any possible associations among variables in the study. SPSS Statistics version 26 (IBM Corp., Armonk, NY, USA) was used for statistical analysis.

Scientific and ethics approval

A declaration of purpose and anonymity guarantee was included and stated clearly in the online survey. Participants were required to confirm their consent before conducting the survey. The study was granted the approval of the research review board of King Saud Bin Abdulaziz University for Health Sciences to ensure compliance with research ethical conduct.

## Results

Descriptive statistics results

**Figure 4:**
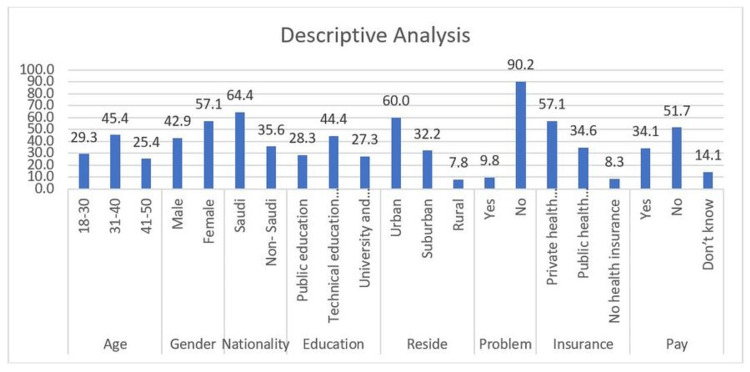
Descriptive analysis.

A total of 205 participants were included in this study after discarding incomplete responses. Table 1 and Figure 4 summarize the demographics of the sample including age, gender, nationality, education, place of residence, health problems, type of insurance, and availability of sick pay. Most participants were in the age group 31-40 with a percentage of 45.4%. Overall, 57.1% of the participants were female. Saudis constituted 64.4% of the participants. Regarding the level of education, 44.4% of the participants held technical education and vocational training degrees. Out of the total respondents, 60% were residents of urban areas.

**Table 1:** Descriptive analysis findings.

		Frequency	Percentage	Valid percentage	Cumulative percentage
Age (years)	18–30	60	29.3	29.3	29.3
	31–40	93	45.4	45.4	74.6
	41–50	52	25.4	25.4	100.0
	Total	205	100.0	100.0	
Gender	Male	88	42.9	42.9	42.9
	Female	117	57.1	57.1	100.0
	Total	205	100.0	100.0	
Nationality	Saudi	132	64.4	64.4	64.4
	Non-Saudi	73	35.6	35.6	100.0
	Total	205	100.0	100.0	
Education	Public education	58	28.3	28.3	28.3
	Technical education and vocational training	91	44.4	44.4	72.7
	University and higher education	56	27.3	27.3	100.0
	Total	205	100.0	100.0	
Area of residence	Urban	123	60.0	60.0	60.0
	Suburban	66	32.2	32.2	92.2
	Rural	16	7.8	7.8	100.0
	Total	205	100.0	100.0	
Health problem	Yes	20	9.8	9.8	9.8
	No	185	90.2	90.2	100.0
	Total	205	100.0	100.0	
Insurance	Private health insurance	117	57.1	57.1	57.1
	Public health insurance	71	34.6	34.6	91.7
	No health insurance	17	8.3	8.3	100.0
	Total	205	100.0	100.0	
Sick pay	Yes	70	34.1	34.1	34.1
	No	106	51.7	51.7	85.9
	Don’t know	29	14.1	14.1	100.0
	Total	205	100.0	100.0	

Voluntary installation (opt-in)

Exploring results for voluntary installation (opt-in) regarding demographic variables, the results showed that out of the 205 participants, 174 intended to install, 28 installed but were intending to install, and the rest 3 were hesitant to install and *May or may not install*, as summarized in Table 2. Out of the 174 participants who intended to install, 133 were less than 40 years of age. Out of those who would not install, 10 participants were less than 40 years of age. Regarding gender, 100 females and 74 males intended to install. Further, 13 males and 15 females intended not to install. Regarding the participants’ nationality, out of 132 Saudi participants, 65 intended to install and 22 intended to not install. On the other hand, out of 73 non-Saudis, 65 intended to install and 6 intended to not install. Regarding the educational level of participants, out of those who intended to install, 46 had public education, 81 had technical education and vocational training, and 47 had university and higher education. Of those who would not install, 12 had public education, nine had technical and vocational training, and seven had university and higher education.

**Table 2:** Voluntary installation (opt-in) intentions in regard to demographics.

Voluntary installation (opt-in)					
Demographics variables		Install	May or may not install	Won’t Install	Total
Age (years)	<40	133	2	18	153
	>40	41	1	10	52
Gender	Female	100	2	15	117
	Male	74	1	13	88
Nationality	Non-Saudi	65	2	6	73
	Saudi	109	1	22	132
Education	Public education	46	0	12	58
	Technical education and vocational training	81	1	9	91
	University and higher education	47	2	7	56
Area of residence	Rural	12	1	3	16
	Suburban	58	0	8	66
	Urban	104	2	17	123
Health problem	No	157	2	26	185
	Yes	17	1	2	20
Insurance	No insurance	15	0	2	17
	Private health insurance	103	2	12	117
	Public health insurance	56	1	14	71
Sick pay	Yes	61	1	8	70
	No	90	2	14	106
	Don’t know	23	0	6	29
News sources	Family and friends	65	1	11	77
	Radio	2	0	0	2
	Social media	64	0	13	77
	Television	43	2	4	49
Social media	Facebook	32	1	7	40
	Instagram	47	1	7	55
	LinkedIn	7	0	3	10
	Twitter	48	0	7	55
	WhatsApp	40	1	4	45
Comprehension level	Yes	115	2	16	133
	No	59	1	12	72
Total		174	3	28	205

According to the participants’ residential area, the majority of participants who intended to install lived in urban areas (104), 58 lived in suburban areas, and 12 lived in rural areas. Exploring the proportion of participants who had health problems, the majority did not have health problems (90.24%). Out of the participants who intended to install, only 17 suffered health problems, and only two of those who did not intend to install had health problems. Regarding the type of health insurance participants had, for those who intended to install, 103 had private health insurance, 56 had public health insurance, and only 15 were not insured. Of those who would not install, 12 had private health insurance, 14 had public health insurance, and only two were not insured. Regarding the question of whether the participants would receive pay if they worked from home, of those who intended to install, 90 participants did not expect any pay working from home, 61 received pay, and the rest (23) were not sure. Of the participants’ who would not install, 14 did not expect any pay working from home, eight received pay, and the remaining six were not sure.

Exploring news sources of participants, for those who intended to install, their news sources were family and friends (65), social media (64), television (43), and Radio (2). Of the participants who would not install, their news sources were social media (13), family and friends (11), and television (4). Exploring the social media usage among participants, for those who intended to install, their used social media platforms were Twitter (48), Instagram (47), WhatsApp (40), Facebook (32), and LinkedIn (7). For participants who would not install, their used social media platforms were Facebook (7), Instagram (7), Twitter (7), WhatsApp (4), and LinkedIn (3).

For the comprehension level of the application, participants were asked three questions about each of the following: the basic permissions that the application requests during the installation, the application message if the person got infected, and the application action if the person was in contact with someone who was infected by the virus. These questions were used to measure individuals’ comprehension levels, where 0 indicates the person does not have a basic comprehension of the Tawakkalna application and 1 indicates a basic level of comprehension.

The comprehension level for participants was high, with 64.88% answering the comprehension questions correctly. Out of the participants who intend to install, 115 had a basic comprehension of Tawakkalna application; for those who would not install, only 16 had a basic comprehension level.

Automatic installation (opt-out)

Exploring results for automatic installation (opt-out) in regard to demographic variables, the results showed that out of 205 participants, 102 intended to keep the application installed after the pandemic, five intended not to, and the rest (98) were hesitant to keep, as summarized in Table 3. Out of the 174 participants who intended to keep, 74 were less than 40 years of age. Out of those who would not keep, five participants were less than 40 years of age. Regarding gender, 56 females and 46 males intended to keep the application.

**Table 3:** Automatic installation (opt-out) intentions in regard to demographics.

Automatic installation (opt-out)					
Demographics variables		Install	May or may not install	Won’t Install	Total
Age (years)	<40	74	74	5	153
	>40	28	24	0	52
Gender	Female	56	59	2	117
	Male	46	39	3	88
Nationality	Non-Saudi	32	41	0	73
	Saudi	70	57	5	132
Education	Public education	29	29	0	58
	Technical education and vocational training	46	42	3	91
	University and higher education	27	27	2	56
Area of residence	Rural	9	7	0	16
	Suburban	31	34	1	66
	Urban	62	57	4	123
Health problem	No	90	90	5	185
	Yes	12	8	0	20
Insurance	No insurance	10	7	0	17
	Private health insurance	59	56	2	117
	Public health insurance	33	35	3	71
Sick pay	Yes	31	36	3	70
	No	57	47	2	106
	Don’t know	14	15	0	29
News sources	Family and Friends	36	40	1	77
	Radio	0	2	0	2
	Social media	39	36	2	77
	Television	27	20	2	49
Social media	Facebook	22	18	0	40
	Instagram	27	26	2	55
	LinkedIn	5	5	0	10
	Twitter	27	27	1	55
	WhatsApp	21	22	2	45
Comprehension level	Yes	61	68	4	133
	No	41	30	1	72
Total		174	102	98	205

Furthermore, three males and two females intended not to keep the application. Regarding participants’ nationality, out of 132 Saudi participants, 70 intended to keep and five intend not to. On the other hand, out of 73 non-Saudis, 32 intended to keep and none did not intend to keep. Regarding the educational level of participants, out of those who intended to keep, 29 had public education, 46 had technical education and vocational training, and 27 had university and higher education. Of those who would not keep, three had technical and vocational training, and two had university and high education. According to participants’ residential areas, the majority of the participants who intended to keep the application lived in urban areas, 31 lived in suburban areas, and nine lived in rural areas. 

Out of the participants who intended to keep, only 12 suffer health problems, and none of those who would not keep had health problems. Regarding the type of health insurance participants had, for those who intended to keep, 59 had private health insurance, 33 had public health insurance, and only 10 were not insured. Of those who would not keep, two had private health insurance, three had public health insurance, and none were not insured. For the question of whether participants would receive pay if they worked from home, of those who intended to keep, 57 participants did not expect any pay working from home, 31 received pay, and the rest 14 were not sure. Of the participants who intended not to keep, two participants did not expect any pay working from home and three received pay.

Exploring news sources of participants, for those who intended to keep, their news sources were social media (39), family and friends (36), and television (27). Of the participants who did not intend to keep, their news sources were family and friends (1), social media (2), and television (2). Exploring the social media usage among participants, those who intended to keep used the following social media platforms: Twitter (27), Instagram (27), Facebook (22), WhatsApp (21), and LinkedIn (5). For participants who intended not to keep, they use the following social media platforms: WhatsApp (2), Instagram (2), and Twitter (1).

For the comprehension level of the application, out of the participants who intend to keep, 61 had a basic comprehension of the Tawakkalna application; of those who did not intend to keep, only four have a basic comprehension level.

Multivariate analysis (multiple logistic regression) results

According to multivariate analysis (multiple logistic regression) results summarized in Table 4, there is no significant impact on the installation and keeping intentions of participants. As a result, the chi-square test of association is used to explore further associations and will be presented in detail in the Discussion section.

**Table 4:** Multivariate analysis (multiple logistic regression) findings. df: degree of freedom; Sig.: significance

Dependent variable	Voluntary installation intention (opt-in)					Dependent variable	Automatic installation intention (opt-out)				
Source	Type III sum of squares	df	Mean square	F	Sig.	Source	Type III sum of squares	df	Mean square	F	Sig.
Age	0.367	2	0.183	1.403	0.248	Age	0.093	2	0.047	0.177	0.838
Gender	0.006	1	0.006	0.049	0.824	Gender	0.146	1	0.146	0.556	0.457
Nationality	0.036	1	0.036	0.276	0.600	Nationality	0.376	1	0.376	1.432	0.233
Education	0.418	2	0.209	1.598	0.205	Education	0.101	2	0.050	0.192	0.826
Area of residence	0.231	2	0.116	0.885	0.414	Reside	0.140	2	0.070	0.265	0.767
Problem	0.003	1	0.003	0.021	0.884	Problem	0.111	1	0.111	0.423	0.516
Insurance	0.430	2	0.215	1.647	0.195	Insurance	0.280	2	0.140	0.533	0.588
Pay	0.070	2	0.035	0.269	0.765	Pay	0.190	2	0.095	0.362	0.697
Trust	0.054	2	0.027	0.206	0.814	Trust	0.036	2	0.018	0.068	0.935

Qualitative analysis results

In this study, the objective of evaluating the contact tracing application Tawakkalna in terms of privacy and security was focused only on permissions analysis and privacy policy analysis due to the Tawakkalna application’s restriction to code access which is required for both run-time permission access analysis (dynamic analysis) and vulnerability analysis (static analysis). The study suggests exploring these latter steps in the availability of the code by the SDAIA in the future.

For permission analysis, through observing the application permission as a Blackbox without going through the code lines in different settings (with internet access, without internet access, updates, etc.). Out of 31 permissions categorized between normal, dangerous, and signature, the Tawakkalna application only requested normal-level permissions: INTERNET, BLUETOOTH, FOREGROUND_SERVICE, and ACCESS_NETWORK_STATE. For privacy policy analysis, by exploring Tawakkalna policies through their website and social media accounts, we concluded that the application fulfilled nine out of 11 privacy principles including privacy policy changes, data retention, third-party sharing, purpose specification, data protection, contact information, app-focused, users’ control, and data collection.

## Discussion

**Figure 5:**
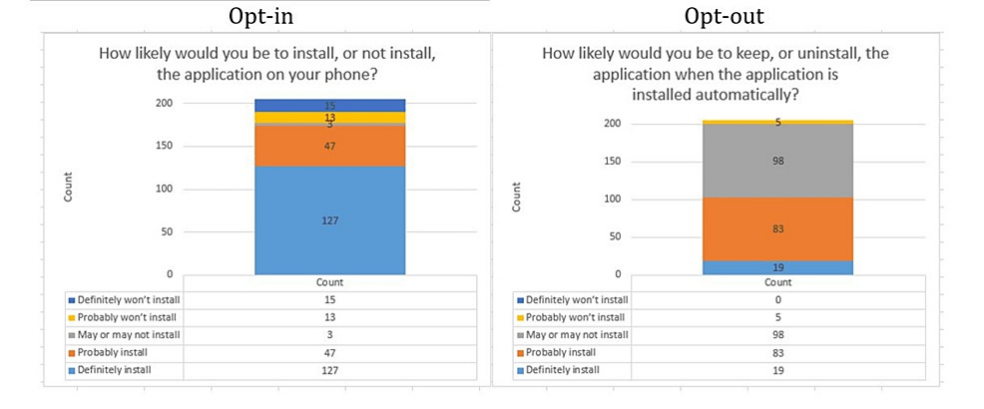
Comparison of opt-in and opt-out installation approaches.

The acceptance of the contact tracing application Tawakkalna is considered high, scoring in the opt-in approach 62% and 23% for the choices definitely install and probably install, respectively. Such support was very low in the opt-out approach which gained merely 9% and 40%, respectively, for the same choices. Figure 5 displays the different responses to these questions in both approaches. The calculated acceptance for the Tawakkalna application under an opt-in approach was 84.87%, and the acceptance under the opt-out approach was 49.75%. The overall support in both approaches was 67.31%.

Comparing the results of this study to a similar study done by Altmann et al. [23] in the UK, Germany, France, Italy, and the US, 74.8% of the respondents accepted a contact tracing application under the opt-in approach and 67.7% supported it under the opt-out approach, with an overall acceptance of at least 68% in each country.

The acceptance for the Tawakkalna application did vary when splitting respondents according to the subgroups of gender, the presence of health problems, nationality, and the probability of getting sick pay or not.

**Figure 6:**
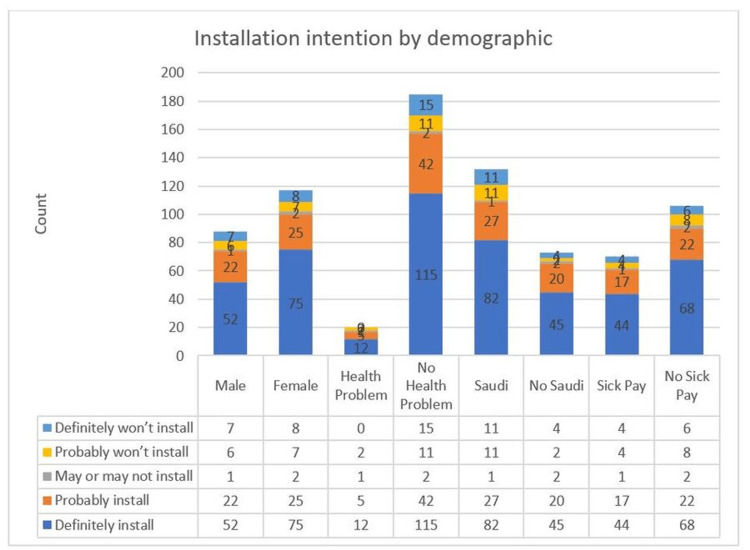
Installation intention by demographics.

The most significant difference appeared when splitting the respondents by the presence of health problems, with 76.58% of acceptance coming from those without health problems and only 8.29% coming from those with health problems such as diabetes, high blood pressure, and heart or breathing problems. The nationality of the respondents came second in acceptance variation with 53.17% support from Saudi nationals and 31.7% to none from Saudis. Regarding the comparison of sick pay to no sick pay, the former scored 29.75% acceptance level compared to 43.9% for the latter. Figure 6 shows the acceptance percentages for different subgroups of respondents. When comparing these results with the study by Altmann et al. [23], we find that there is no significant variation in the acceptance levels for the counties included in the study.

**Figure 7:**
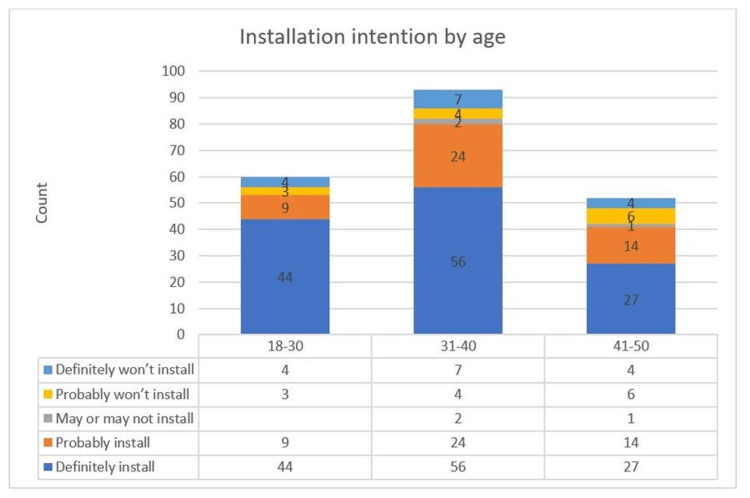
Installation intention by age.

On the other hand, splitting respondents by age did not have a significant variation in acceptance levels. Results showed that the age group 31 to 40 had the highest acceptance for the application, which could be a result of advanced maturity compared to the age group 18 to 30, which came second in acceptance level, and being a little bit more technologically savvy than the age group 41 to 50, which came last [29]. Figure 7 shows acceptance comparisons according to age groups. These results are similar to those reported by Altmann et al. [23] for the UK, Germany, France, Italy, and the US.

**Figure 8:**
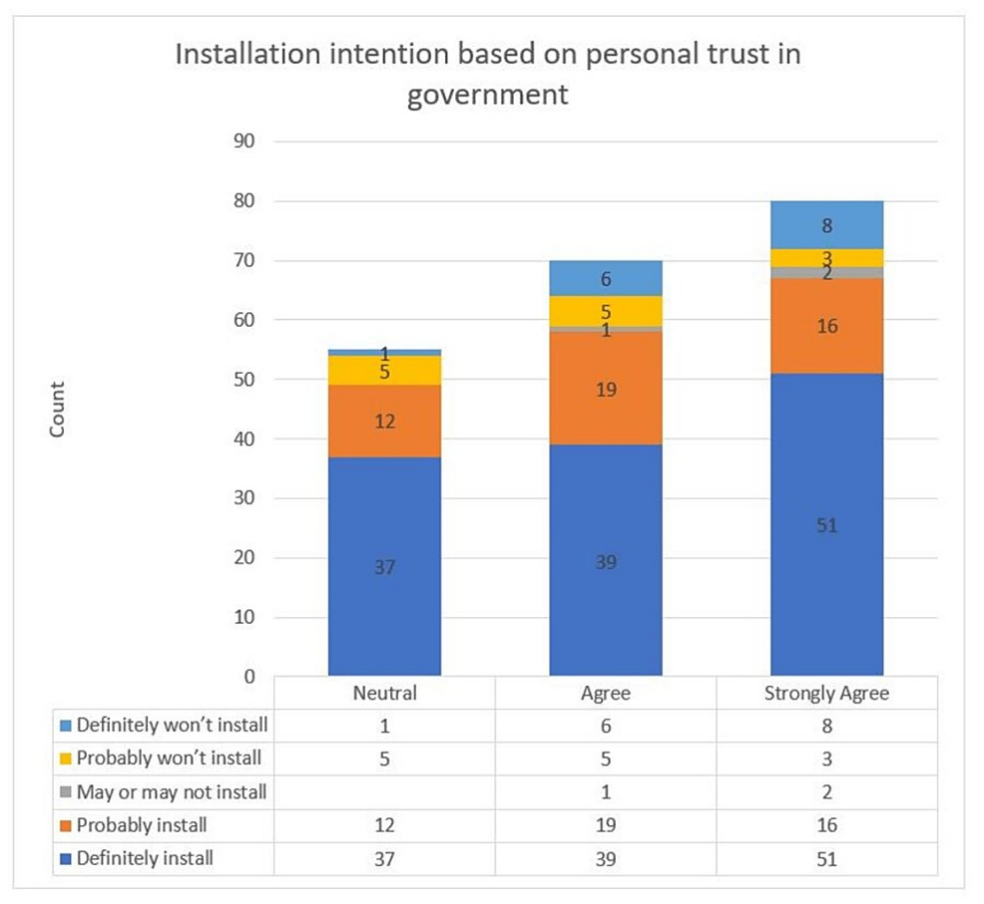
Installation intention based on personal trust in the government.

The only significant variation in contact tracing applications acceptance level in the Altmann et al. study [23] occurred when the participants were split by their trust level in the government to do the right thing. According to this study, people who had more trust in the government were more likely to install contact tracing applications. In our study, those with high trust levels in the government and who were willing to install the application constituted 60.98% of the participants, and the number reduced significantly for those with less trust in the government at 23.9%. Figure 8 shows the acceptance levels for the application according to government trust.

**Figure 9:**
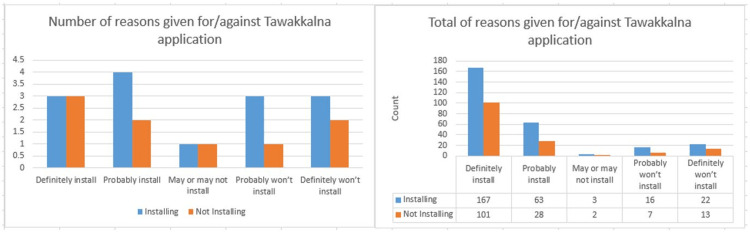
Analysis of reasons given for/against the installation of the Tawakkalna application.

Figure 9 shows that participants had more reasons to install the application than the issues against its installation. This is even true in the case of those choosing probably or definitely would not install the application. The number of reasons to install the application decreases as participants become less committed to installation. Figure 9 also shows that, even though participants chose to install the application, they still chose at most three reasons against its installation. This could be a result of not fully understanding the application to individuals and their communities. The findings of this study are similar to that of Altmann et al. [23].

**Figure 10:**
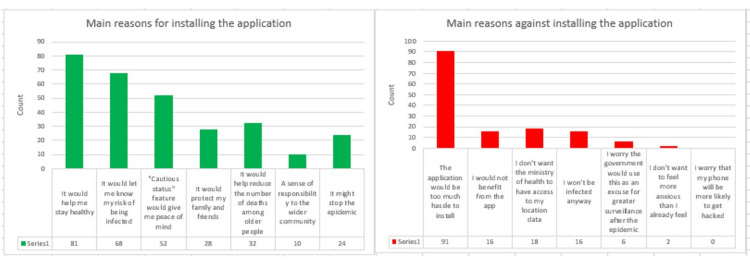
Analysis of reasons for/against the installation of the Tawakkalna application.

The most common reasons chosen for supporting or being against the application installation are presented in Figure 10. The most common reasons for supporting the application installation were self and surrounding individual protection focused, it would help me stay healthy, it would let me know my risk of being infected, and the *cautious status* feature would give me peace of mind.

On the other hand, the most common reason against installing the application was that *The application would be too much hassle to install*, which is an indication that more efforts need to be put into finding new ways to promote how easy it is to install and use the application. It is notable that the second common reason against the application installation was *I don’t want the ministry of health to have access to my location data*. In the study by Altmann et al. [23], the reasons against installing the application were fear of surveillance and security issues at 42 and 35, respectively.

**Figure 11:**
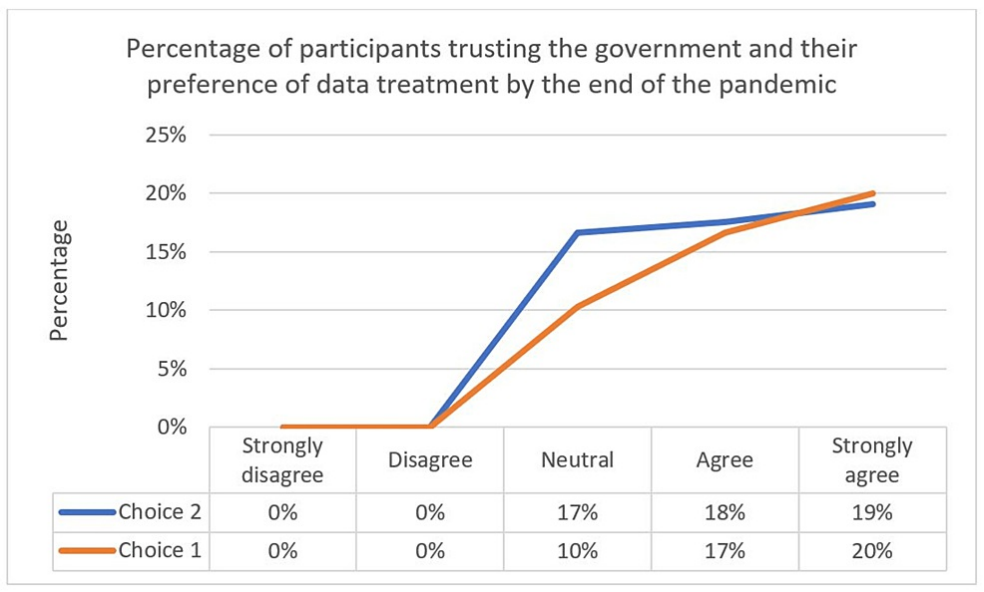
Percentage of participants trusting the government and their preference for data treatment by the end of the pandemic.

When participants were asked to choose their preference on what to do with the collected data, there were no significant differences between those who chose to de-identify the data and make it available for research and those who chose to have the collected data automatically deleted when the pandemic ends. Figure 11 shows the percentages of each choice.

**Figure 12:**
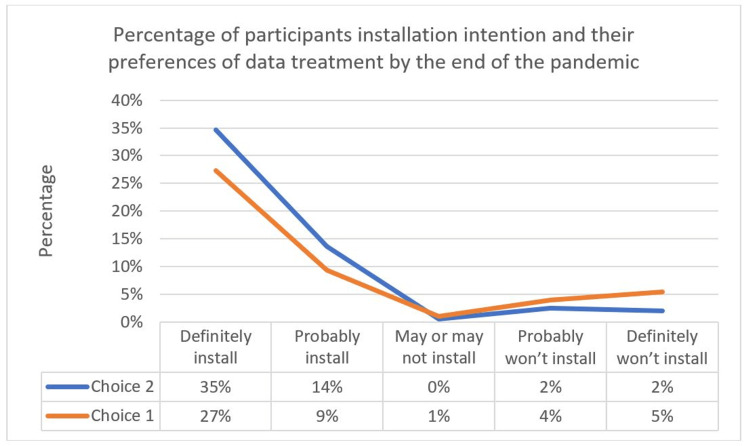
Percentage of participants’ installation intention and their preferences for data treatment by the end of the pandemic. Choices 1 and 2 indicate *Delete data automatically when the pandemic ends* and *De-identify the data and make it available for research*, respectively.

Those who supported the application installation preferred to have the collected data de-identified and made available for research, with 49% of all the participants, and 36% chose the option to have the collected data automatically deleted by the end of the pandemic, as presented in Figure 12. Those who chose not to install the application also had a preference for having the data de-identified and made available for research, and 9-4% wanted the data to be automatically deleted by the end of the pandemic. Compared to the Altmann et al. study [23], 59.9% of the participants in all five countries preferred to have the data de-identified and made available for research. The study also found that participants who were less likely to download the application were not in favor of their data being available for research after de-identifying it, and there was no significant difference in choices between those with high and low trust in the government, which is similar to our study findings.

**Figure 13:**
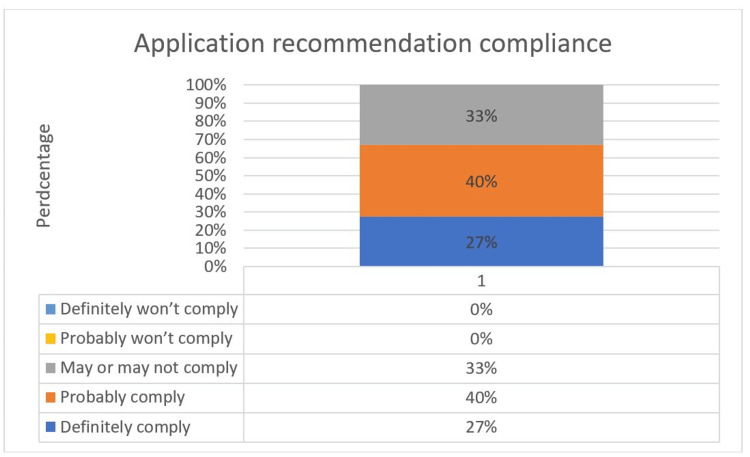
Application recommendation compliance.

When asked how likely would they be to comply with the recommendation of the application to self-isolate at home for 14 days if they had been in close contact with an infected person, 67% of the participants answered in favor of compliance. Figure 13 shows the answers to this question. Comparing this to the Altmann et al. study [23], 96% of the participants from the five countries involved in the study answered in favor of compliance.

**Figure 14:**
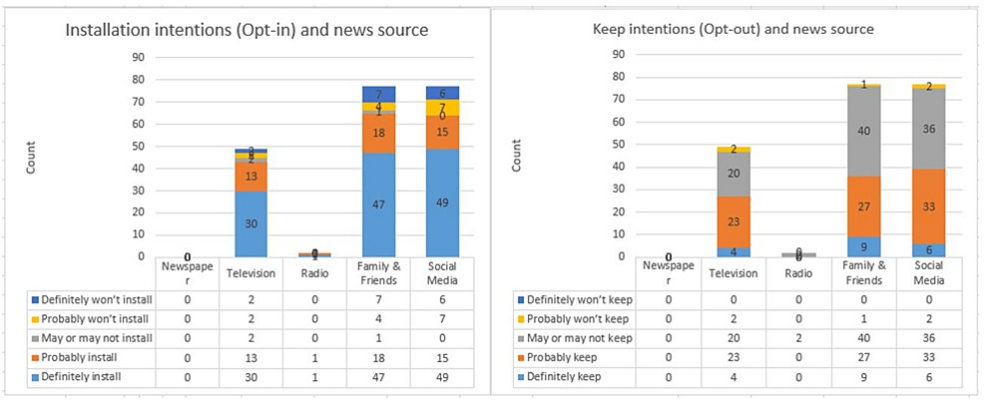
Analysis of news sources.

Regarding participants’ most popular news sources, for those who intended to install the Tawakkalna application, news sources included family and friends (65), social media (64), and television (43). The same applied to participants willing to keep the application if automatically installed in regard to most popular news sources, i.e., family and friends (36), social media (39), and television (27). Figure 14 summarizes these results.

Exploring the association of willingness to install the application with other factors, we found a strong positive linear association (p = 0.033, r = 0.325) with whether the government should ask mobile phone providers to install the application on all phones. People who chose to definitely install or probably install were more supportive of this action at 62% (127 out of 205).

A strong association existed between the two factors age and education, and the act of compliance with the application’s recommendations to self-isolate for 14 days if infected or needed. For the age factor (p = 0.002, r = 0.06), 66.8% of the participants chose to comply, and from this percentage, 41.6% were between 18 and 40 years of age. On the other hand, for the education factor (p = 0.00, r = 0.022), 14.63% of the participants choose to comply, and out of these, 80% had higher education.

Sparing people from the lockdown if they had the status immune on their phones seemed to be a drive to install the application and this was associated with the education (p = 0.042, r = 0.093. 86.34%) of the participants who chose to install the application, and from this percentage, 74.58% had higher education.

The question exploring participants’ preferences on how to deal with the collected data once the pandemic is over had associations with age, gender, and nationality. For age (p = 0.004, r = 0.2), the number of participants choosing to automatically delete the collected data was similar in all age groups, while 55% of those choosing to de-identify the data and make it available for research were in the age group 31 to 40 years old. The association with gender (p = 0.000, r = 0.000) was 69.32% of males choosing to have the collected data de-identified and made available for research, and 58.97% of females choosing to automatically delete the collected data once the pandemic is over. For the nationality factor (p = 0.017, r = 0.017), choices of whether to delete the collected data or de-identify it and make it available for research scored 53% and 46.97%, respectively, between Saudi participants and 35.62% to 64.38, respectively, between non-Saudi participants.

Exploring the association between the preferred approach of installation (opt-in or opt-out), and whether participants get sick pay or not (p = 0.04, r = 0.098), 67.32% of the participants chose the automatic installation (opt-out). Out of these, 45.65% did not get sick pay. Out of the 32.68% who chose the optional installation (opt-in), 64.18% did not get sick pay.

For permission analysis, compared to other contact tracing applications in the study [24], 31% of the permission requested were within the dangerous category, which creates direct threats to users’ safety. Furthermore, 66% of the permission requests were considered normal, and 3% of requests were signature requests. The study found that Gerak (Malaysia) application requested eight dangerous permissions out of 18, while Mahakavach (India) requested seven out of 14. The Tawakkalna application only requested four normal permissions, which is comparable to Stopp Corona (Austria) which was the only application that did not ask for dangerous permissions.

For privacy analysis, the Tawakkalna application fulfilled nine out of 11 privacy principles, which indicates that it reached the highest level of compliance with privacy principles, landing before Gerak (Malaysia) which fulfilled eight out of the 11 privacy principles.

Limitations and future suggestions

A limitation of this study is that the online sample (N = 205) recruited did not meet the calculated sample size of 377, which may not fully represent the entire population of Saudi Arabia. Participants’ willingness to share their views through this survey, which is not considered a formal channel, may have affected the general response. If the survey was administered through the Tawakkalna application, this might create a much better perceived response from the public. 

Regarding future consideration, we recommend repeating the study because the Tawakkalna application has a more mature level and will surely include other important features in the future. Moreover, research is essential to examine how the topic of the Infodemic has affected the installation intentions of the Tawakkalna application.

## Conclusions

This study finds high support for the Tawakkalna contact tracing application among all groups with different ages, genders, nationalities, education levels, places of residence, health insurance, and health problems (diabetes, high blood pressure, heart or breathing problems). Although the high acceptance is encouraging, many have concerns regarding privacy and trust. These are considered the main factors affecting acceptability. Acceptance of contact tracing applications depicted a positive association between individuals’ trust in the government and their willingness to install the application.

In Saudi Arabia, people have a strong perspective on the benefits of installing the Tawakkalna application to reduce the risks of COVID-19 infection and curb the spread of the virus.

Participants in Saudi Arabia have high compliance with the application recommendation due to their belief that Tawakkalna will keep them informed about the risks of COVID-19 and will help them distance themselves from possible infections. The Tawakkalna application scored high compared to other countries in regards to population willingness to install the application. This is because the government of Saudi Arabia delegated the development and control of the Tawakkalna application to a highly transparent and open authority, the SDAIA. In return, SDAIA, reached out to the population to gain their trust through the most used outlets. In contrast, other countries failed to address privacy and security policies followed by the contact tracing application, especially in Germany and Sweden. Furthermore, regarding transparency with users on how the collected data will be treated, the study found that nearly 53% chose to de-identify their data and allow the data to be available for research. Almost 61% of the participants stated that they trusted the government to do what was right in regard to the type of installation (opt-in or opt-out), as well as with the collected data.

For privacy and security investigation’s results were evidence of the maturity of the Tawakkalna application compared to other contact tracing applications in other countries. Some of these applications were engineered fast in the early phases of the pandemic. The investigation did not assess the application’s code quality due to access limitations. The Tawakkalna application demonstrated a high-quality solution in regard to the provided privacy policies, the requirement of dangerous invasive permission access patterns, and compliance with privacy principles. Static and dynamic code analysis to assess vulnerabilities is suggested for future considerations.

Although it is difficult to trace the installation rates of the Tawakkalna application from the beginning of its launch till now, and the drivers behind these rates, the Saudi government’s efforts to meet the factors mitigating rejection and increasing the acceptance levels are very clear.
